# Design of a Janus Composite Patch with Anti-Adhesive and Growth-Promoting Functions for Abdominal Wall Defect Repair

**DOI:** 10.3390/bioengineering12050522

**Published:** 2025-05-14

**Authors:** Qingxi Hu, Xiaoyang Hou, Hekai Shi, Yongteng Song, Bing Zhou, Xinli Hu, Haiguang Zhang, Yan Gu

**Affiliations:** 1 Rapid Manufacturing Engineering Center, School of Mechatronical Engineering and Automation, Shanghai University, Shanghai 200444, China; 2 National Demonstration Center for Experimental Engineering Training Education, Shanghai University, Shanghai 200444, China; 3 Shanghai Key Laboratory of Intelligent Manufacturing and Robotics, Shanghai University, Shanghai 200444, China; 4 Shanghai Medical College of Fudan University, Fudan University, Shanghai 200032, China

**Keywords:** abdominal wall defects repair, Janus, electrospinning, electrospray

## Abstract

Tension-free hernioplasty has effectively reduced postoperative recurrence and mitigated complications by employing polymer patches. However, clinically used polymer patches often fall short in terms of the anti-deformation, anti-adhesion, and tissue integration functions, which can result in visceral adhesions and foreign body reactions after implantation. In this study, a Janus three-layer composite patch was developed for abdominal wall defect repair using a combination of 3D printing, electrospraying, and electrospinning technologies. On the visceral side, a dense electrospun polyvinyl alcohol/sodium hyaluronate (PVA/HA) scaffold was fabricated to inhibit cell adhesion. The middle layer, composed of polycaprolactone (PCL), provided mechanical support. On the muscle-facing side, a loose and porous electrospun nanofiber scaffold was created through electrospraying and electrospinning, promoting cell adhesion and migration to facilitate tissue regeneration. Mechanical testing demonstrated that the composite patch possessed excellent tensile strength (23.58 N/cm), surpassing the clinical standard (16 N/cm). Both in vitro and in vivo evaluations confirmed the patch’s outstanding biocompatibility. Compared with the control PCL patch, the Janus composite patch significantly reduced the visceral adhesion and enhanced the tissue repair in animal models. Collectively, this Janus composite patch integrated anti-deformation, anti-adhesion, and tissue-regenerative properties, providing a promising solution for effective abdominal wall defect repair.

## 1. Introduction

Abdominal wall defects are a common surgical disease caused by weakened, broken, and leaking abdominal muscles, etc. [1]. Reports indicate that at least 20 million abdominal wall hernia surgeries are performed worldwide annually [2]. Compared to traditional suture hernia repair, which has a high recurrence rate, tension-free hernioplasty lowers postoperative recurrence and mitigates complications to some extent by implanting a polymer patch [3,4,5]. Conventional polypropylene (PP), polyethylene terephthalate (PET), and polytetrafluoroethylene (PTFE) patches are widely used in tension-free hernioplasty due to their superior mechanical strength and stability [6]. However, the non-degradability and limited biocompatibility of these polymer patches can result in foreign body reactions, abdominal adhesions, and poor wound healing following implantation, which may lead to long-term complications such as intestinal obstruction and infertility in female patients [7,8,9,10]. Therefore, an ideal biological patch should have not only excellent biocompatibility, degradability, and mechanical strength but also the ability to prevent postoperative adhesions and promote wound healing [11].

Currently, researchers have proposed various strategies to enhance the performance of polymer patches. Applying physical coatings to PP patches, such as modified chitosan, has become a mainstream approach to effectively isolate the patch from visceral tissue and prevent postoperative adhesions [12]. However, these polymer-based composite patches may lead to poor healing and the corresponding inflammatory reactions due to the lack of a microenvironment suitable for cell migration and growth [13]. Hence, patches need to be multifunctional, not only in terms of the mechanical properties but also in terms of preventing adhesions on the visceral side and promoting wound healing on the muscular side. To address these challenges, Janus structures that can harmoniously integrate opposite properties have gained significant attention. For instance, Tang et al. developed a Janus composite porous hydrogel featuring a loose, porous small intestinal submucosa (SIS) layer that accelerates tissue healing, while a dense porous polyvinyl alcohol (PVA) layer with embedded exosomes prevents visceral adhesion and maintains mechanical strength [14]. Based on the above analysis, this research attempted to exploit a Janus three-layer composite patch, comprising a growth-promoting layer on the muscle-fitting side to provide a microenvironment suitable for cell migration and wound healing, a middle support layer to ensure sufficient mechanical properties, and an anti-adhesion layer on the organ-facing side to prevent postoperative visceral adhesions.

Electrospun fiber scaffolds are widely used in hernia repair due to their high porosity and three-dimensional interconnected structure being similar to natural extracellular matrix (ECM) [15,16]. The pore size of electrospun fiber scaffolds is about 200–1000 nm, which can effectively prevent cell migration and infiltration while allowing for efficient nutrient exchange [17,18]. For example, Carmen Chalony et al. used poly(ethyl-2) cyanoacrylate and polyurethane electrospun scaffolds to reduce the bioadhesion of the patch [19]. Meanwhile, electrospinning technology enables the combination of biomaterials and polymers to create electrospun membranes with enhanced mechanical properties and biocompatibility. For example, Rui Gao et al. combined PLGA (poly(lactic-co-glycolic acid)) and chondroitin sulfate for electrospinning to ensure the mechanical properties while reducing the local inflammatory response [20]. However, the small pore size of electrospun fiber scaffolds also limits their application in the field of biomedical engineering, as cell diameters typically exceed 5 μm, which makes them almost impervious in dense electrospun fiber scaffolds. Hence, to improve the pore size of electrospun fiber scaffolds, techniques such as sacrificial salt and direct laser processing have been proposed to achieve larger pores while maintaining the structural integrity of the scaffolds [21,22]. However, these methods are limited by high costs and complex processes. In contrast, combining electrospraying produces electrospun fiber scaffolds with a uniformly dispersed macroporous structure at a lower cost, ensuring structural integrity while providing a suitable microenvironment for cell growth and migration [23]. Electrostatic spraying was used to eject soluble microspheres and randomly embed them layer by layer into the electrospun nanofiber scaffolds. Subsequently, the microspheres were removed by a washing process to form randomly distributed micropores in the fibrous membrane, which altered the porosity characteristics of the scaffolds.

For multilayer abdominal wall repair patches, the selection of an appropriate material for each layer plays an indispensable role in the performance of the patch. Polycaprolactone (PCL) is widely used in biomedical applications due to its good stability, mechanical properties, and flexibility [24,25]. By serving as a support layer material, PCL contributes to the stability of the Janus three-layer composite patch. However, the main disadvantage of PCL is its low tissue affinity and hydrophobicity [26]. Polyethylene oxide (PEO) is known for its excellent hydrophilicity and is a commonly used synthetic polymer for electrospinning. Furthermore, PEO can effectively reduce the surface tension of the spinning solution, promoting a more uniform fiber diameter distribution. However, its rapid in vivo dissolution limits its practical application [27]. Thus, the electrospun scaffolds made from a PCL/PEO combination exhibit excellent stability and biocompatibility, making them suitable materials for the growth-promoting layer of the patch. For the anti-adhesive layer, HA is a naturally biodegradable biomaterial that can reduce the inflammatory response, promote tissue repair and inhibit postoperative bleeding [28]. Studies have demonstrated that HA hydrogel effectively reduces adhesion without adversely affecting wound healing [29]. However, HA is only soluble in water, but its aqueous solution is difficult to electrospin. Even at a low concentration, an HA aqueous solution is highly viscous, which limits its usable concentration for electrospinning and prevents the formation of a stable fiber structure [30]. With superior anti-adhesion properties and excellent film-forming capabilities, PVA is an ideal choice for preparing electrostatic spinning scaffolds in combination with difficult-to-spin water-soluble biomaterials [31,32]. For example, Hu Q et al. combined PVA with soy peptide (SP) to prepare electrospun nanofiber scaffolds that demonstrated effective film formation and strong anti-adhesion properties [33]. Thus, preparing PVA electrospun nanofiber scaffolds containing HA as anti-adhesion layers for patches via electrospinning is feasible.

Therefore, in this study, a Janus three-layer composite patch was prepared based on 3D printing, electrospinning, and electrospraying. The dense and porous anti-adhesion layer (PVA/HA) was prepared via electrospinning technology; the loose and porous growth-promoting layer (PCL/PEO) was prepared by the combination of electrospinning and electrospraying; and the PCL was prepared as a support layer via 3D printing technology. For the constructed asymmetric Janus three-layer composite patch, the loose and porous growth-promoting layer can promote cell adhesion, migration and growth, the dense and porous anti-adhesion layer can effectively prevent visceral adhesion, and the intermediate support layer can provide stable mechanical strength and deformation resistance, thus realizing effective abdominal wall defect repair.

## 2. Materials and Methods

### 2.1. Materials

Miracll Chemicals Co., Ltd. (Yantai, China) supplied the PCL (Mn = 80,000), while the PVA (1799), PVA (0588), and HA (95%, source: *Streptococcus equi*) were obtained from Macklin (Shanghai, China). Additionally, the PEO (Mw: 600 kDA), N,N-dimethylformamide (DMF), dichloromethane (DCM), and acetic acid (AA) were procured from Sinopharm Chemical Reagent Co., Ltd. (Shanghai, China).

### 2.2. Preparation of Biomaterial Inks

To prepare the support layer, an 85% *w*/*v* PCL solution was obtained by dissolving PCL in DCM.

In order to create a 12% *w*/*v* PCL/PEO solution for the growth-promoting layer, PCL was dissolved in a combination of DCM and DMF (7:3, *v*/*v*) in a 9:1 *w*/*w* ratio with PEO for 30 min. To create a PVA (0588) solution with a variety of concentrations, PVA (0588) was dissolved in deionized water and heated at 95 °C for two hours. Upon cooling, the resulting PVA (0588) solution was prepared with a water-to-AA ratio of 6:4.

To prepare the anti-adhesive layer, PVA (1799) (10% *w*/*v*) was dissolved in deionized water at 95 °C. The PVA/HA solution was created by dissolving HA in the PVA (1799) solution at a ratio of 0.8% *w*/*v* after it had cooled to room temperature.

### 2.3. Manufacturing of Patch

**Figure 1:**
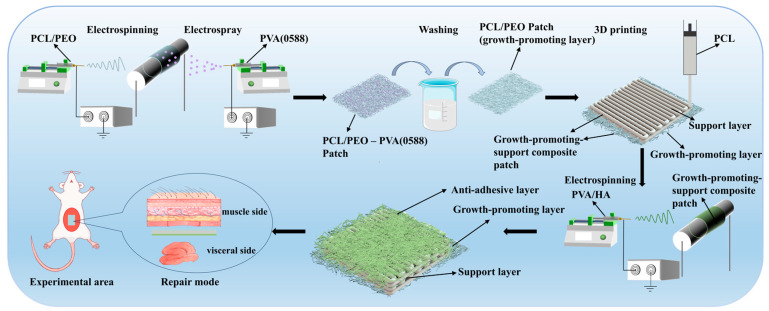
Schematic illustration of preparing the composite patch.

The preparation process for the Janus three-layer composite patch is shown in Figure 1, and all the preparation processes were carried out at room temperature.

The growth-promoting layer of the patch was first prepared, which was synchronized by a combination of electrospinning and electrospray technology. For the electrospinning, the prepared PCL/PEO solution was loaded into a 10 mL syringe with a 22-gauge metal needle and delivered at a constant rate of 0.6 mL/h via a syringe pump. A high voltage of 13 kV was applied at the needle to form continuous nanofibers and the distance between the needle and the drum was set to 15 cm. For the electrospraying, different concentrations (3%, 6%, 9%, and 12% *w*/*v*) of PVA (0588) solution were loaded into a 10 mL syringe with a 20-gauge metal needle on the other side, a high voltage of 15 kV was applied, the distance between the needle and the drum was set to 15 cm, and electrospraying was carried out at four feed rates (0.5, 1, 2, and 4 mL/h), respectively. Fibers and microspheres were deposited synchronously on a rotating drum collector. The preparation of the composite scaffolds lasted for 8 h, and the PCL/PEO–PVA (0588) patch was obtained. After the preparation of the PCL/PEO–PVA (0588) patch, it was immersed in deionized water to remove the embedded PVA (0588) microspheres. The water was changed every 8 h for 24 h to ensure adequate dissolution of the microspheres. Finally, the patch was dried in a drying oven to obtain structurally stable and pore-rich PCL/PEO patches, which were the growth-promoting layer of the Janus three-layer composite patch.

After the growth-promoting layer was prepared, the support layer of the patch was constructed on its surface using 3D printing technology. The process was as follows. Firstly, the prepared PCL solution was injected into a print cartridge equipped with a 22-gauge needle, and the air pressure required for extrusion was provided by a compression pump. Subsequently, the cylinder was mounted on a three-axis motion control platform and the print path was programmed for material deposition. The print path was a square grid structure with 2.5 mm grid cells and 4 layers. During the printing process, the moving speed of the cylinder was set to 6 mm/s, and the air pressure was kept at 0.4 MPa. After the process, the growth-promoting–support composite patch was obtained.

After the growth-promoting–support composite patch was prepared, the anti-adhesion layer was constructed on the other side of the support layer by electrospinning. The electrospinning process was used as follows. The growth-promoting-support layer was attached to the drum in such a way that the growth-promoting layer was pressed against the drum and the support layer was facing outward. The PVA/HA solution then was loaded into a 10 mL syringe with a 20-gauge metal needle, and the solution was delivered at a constant rate of 0.4 mL/h by a syringe pump. A high voltage of 10.5 kV was applied at the needle and the distance between the needle and the drum was set to 10 cm. The entire electrospinning process lasted 10 h. After the preparation, the complete Janus three-layer composite patch was obtained.

### 2.4. Micromorphology Characterization

The samples were coated with a layer of gold with a thickness of about 5 nm, coated with a sputtering current of 15 mA and a sputtering time of 50 s. The microstructure of the patches was then analyzed using a scanning electron microscope (SEM) (Hitachi, Ltd., Shanghai, China) at an accelerating voltage of 15 kV, with a current of 120 μA during the SEM imaging. Using ImageJ (1.54f) software to measure 50 randomly chosen fibers and compute the average, the mean nanofiber diameter was found.

### 2.5. Pore Size and Porosity Test

The test samples were cut into squares of 20 mm × 20 mm, and the thickness of the electrospun scaffolds was measured at five randomly chosen points using an electronic digital OD micrometer to determine the average thickness of each sample. The mass of the scaffolds was measured using an electronic balance and the average fiber diameter was verified using a cross-sectional SEM. Using the following formula, the average density of the electrospun scaffolds ($\rho_{\mathrm{fiber}}$) was determined:

(1) $$\rho_{\mathrm{fiber}}=\frac{m_{\mathrm{fiber}}}{h_{\mathrm{fiber}}l_{\mathrm{fiber}}^{2}}$$

For the electrospun scaffolds, $m_{\mathrm{fiber}}$ is the mass of the scaffold, $h_{\mathrm{fiber}}$ is the average thickness of the scaffold and $l_{\mathrm{fiber}}$ is the side length of the square sample.

The following formula was used to determine the electrospun scaffolds’ porosity [34]:

(2) $$\varepsilon \;=\frac{\rho_{0}-\rho }{\rho_{0}}\;\times \;100\%$$

For the electrospinning and electrospraying, $\rho_{0}$ is the average density of the material and ρ is the apparent density of the electrospun scaffolds.

Using the following formula, the average density of the mixture materials was determined [34]:

(3) $$\frac{1}{\rho_{0}}\;=\frac{\omega_{\mathrm{fiber}}}{\rho_{\mathrm{fiber}}}+\frac{\omega_{\mathrm{PVA}}}{\rho_{\mathrm{PVA}}}$$

where $\omega_{fiber}$ and $\omega_{\mathrm{PVA}}$ are the weight fractions of PCL/PEO and PVA (0588), respectively, while $\rho_{\mathrm{fiber}}$ and $\rho_{\mathrm{PVA}}$ are the densities of PCL/PEO and PVA (0588), respectively.

The following formula was used to obtain the electrospun scaffolds’ average pore radius (r):

(4) $$r\;=\frac{\sqrt{\pi }}{4}(\frac{\pi }{2\log\left(\frac{1}{\varepsilon }\right)}-1)d$$

where ε and d represent the porosity and average fiber diameter of the electrospun scaffolds, respectively [35].

### 2.6. Fourier Transform Infrared Spectroscopy (FTIR) Analysis

Infrared spectroscopy analysis was conducted on the PVA, HA, PVA/HA, PCL, PEO, and PCL/PEO using a Fourier transform infrared spectrometer. The experimental conditions included a spectral resolution of 2 cm^−1^, and a wavelength range of 4000 to 500 cm^−1^.

### 2.7. Wettability Analysis

Using a goniometer and a previously outlined process, the water contact angle (WCA) was measured to determine the materials’ hydrophilicity [36]. The patches were trimmed to 20 × 20 mm^2^ before testing. Each patch received a 4 μL drop of deionized water, and a goniometer was used to determine the water contact angle.

### 2.8. Mechanical Property Evaluation

A universal testing machine (Songdun Machine Equipment, Shanghai, China)was used to measure the patches’ tensile strength. With a few modifications, the experiments were carried out in accordance with ASTM D5035-2011 [37]. ASTM D5035-2011 is an international standard for determining the breaking strength and elongation of textile fabrics by the strip method for evaluating the mechanical properties of materials. Before the test, the patch was cut into 25 mm × 25 mm size. To avoid slippage of the wetted patch due to an excessive stretching speed, the stretching test was performed at a speed of 5 mm/min (standard 30 mm/min) until the patch broke. The universal testing machine reads the corresponding load and tensile elongation and generates the corresponding data. Three samples were tested in each group. The following formula was used to obtain the patch’s tensile strength N:

(5) $$N\;=\frac{F}{L_{0}}$$

where F is the applied load and $L_{0}$ is the length of the patch perpendicular to the stretching direction [38].

### 2.9. In Vitro Cell Experiments

#### 2.9.1. Cell Culture

An endothelial cell growth medium including 90% high-glucose DMEM, 10% fetal bovine serum, and 1% penicillin–streptomycin was used to cultivate human umbilical vein endothelial cells (HUVECs). The cells were kept in a humidified incubator with 5% CO_2_ at 37 °C. After the confluence on the culture plate reached 70–80%, the HUVECs were passaged, and cells from passages 5–8 were utilized for the in vitro tests.

#### 2.9.2. Adhesion Analysis

Under the condition of light protection, 1.5 mL of buffer was placed in a 2 mL centrifuge tube. Next, 1 μL of calcein and 3 µL of propidium iodide were added to the centrifuge tube and shaken well to make a thorough mixture to obtain the cell-staining solution. The air-dried patches were first sterilized by soaking them in 75% medical alcohol for 4 h. Afterward, they were washed three times with phosphate-buffered saline (PBS) and soaked in PBS for an additional 4 h to eliminate any residual alcohol. Following this, the patches were placed in cell culture serum-containing medium for 4 h. A suspension of HUVECs at a density of 1.0 × 10^5^ cells/mL was then carefully seeded onto the patches, ensuring complete surface coverage. At 1, 3, and 5 days post-incubation, the patches were removed, and 80 µL of a live/dead cell staining solution was uniformly applied under dark conditions. The samples were incubated for 10 min at a constant temperature, and the attached live and dead cells on the patch surfaces were observed using an inverted fluorescence microscope. After obtaining the cell live/dead-stained images, the cell coverage was calculated using the software Image J.

#### 2.9.3. Cell Proliferation Assay

Scaffold leachate was prepared by soaking the sterilized scaffolds in serum-containing medium for 48 h. Then, HUVECs were inoculated into 96-well plates at a density of 1 × 10^5^ cells/mL. After incubation for 4 h to allow complete cell attachment, the original medium was removed and 100 μL of scaffold leachate and fresh serum-containing medium (positive control) were added to each well. After 1, 3, and 5 days of incubation, the leachate was removed and 100 μL of serum-free medium with 10 μL of CCK-8 reagent was added to each well, followed by 1 h of incubation. The absorbance at 450 nm was measured using a microplate reader to assess the metabolic activity of the cells.

#### 2.9.4. Cell Morphology Assay

The cytoskeleton and nucleus were stained with fluorescence to visualize the cell morphometry. Following three days of HUVEC culture on the patches, DAPI was used to stain the nuclei and tetramethylrhodamine (TRITC)–phalloidin to stain the cytoskeleton. The cells on the patches were first fixed for 10 min in 4% paraformaldehyde, and then they were given two PBS washes. After five minutes of exposure to a membrane disruption solution, the cells underwent two PBS washes. After 30 min of incubation with a 100 nM phalloidin solution, the patches were rinsed twice with PBS. After 30 s of DAPI staining of the nuclei, the stained samples were examined using an inverted fluorescence microscope.

### 2.10. In Vivo Animal Experiment

Male Sprague–Dawley rats weighing between 180 and 200 g (3 rats per group) were used for the experiment. The rats were obtained from the Animal Center of Huadong Hospital. Anesthesia was induced by injecting 10% chloral hydrate into the rats’ abdomens under sterile conditions. The abdominal area was then cleaned with 75% alcohol. A 15 × 15 mm^2^ defect was created along the abdominal midline, and a 20 × 20 mm^2^ patch was placed over the defect. The defect site was closed using sutures. The patches were surgically removed at two different time points: 2 and 4 weeks post-surgery.

Prior to tissue collection, the rats were euthanized in a CO_2_ chamber in accordance with the approved animal protocol. A macroscopic assessment was then performed to evaluate the adhesion. For additional pathological examination, the abdominal wall—including the defect area—was removed and preserved in 10% paraformaldehyde. Three staining methods were used to analyze the tissue regeneration at the defect site: CD31, Masson’s trichrome, and hematoxylin–eosin (H&E). The Animal Research Committee of Huadong Hospital, affiliated with Fudan University, gave its permission for all the animal-related procedures (approval number: 2023-HDYY-32JZS).

### 2.11. Statistical Analysis

All the experimental results were expressed as the mean ± standard deviation (SD). The statistical analyses were performed using the software GraphPad Prism (9.5) with the one-way analysis of variance (ANOVA) method. The differences of * *p* ≤ 0.05, ** *p* < 0.01 and *** *p* < 0.001 were considered statistically significant.

## 3. Results

### 3.1. Micromorphology Characterization and Pore Size and Porosity Test

**Figure 2:**
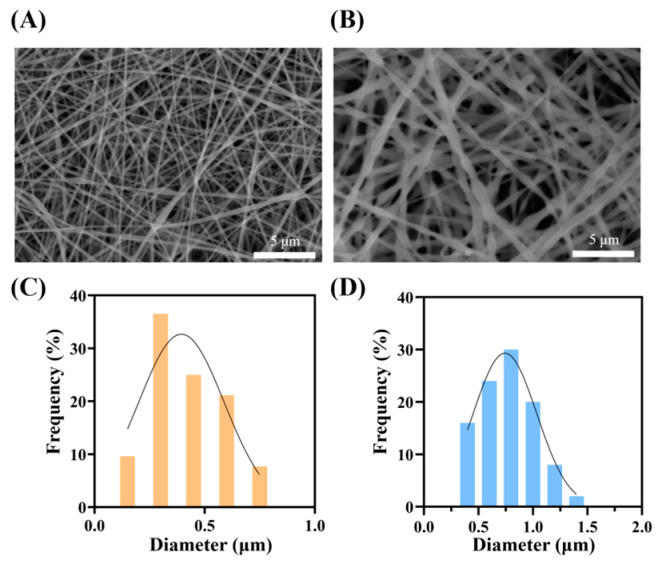
The SEM image of the PVA/HA patch (**A**) and PCL/PEO patch without electrospraying (**B**). The diameter distribution of the PVA/HA patch (**C**) and PCL/PEO patch without electrospraying (**D**).

The prepared PVA/HA and PCL/PEO microscopic morphology characterization results are shown in Figure 2. According to the SEM images, the electrospun fibers exhibit a consistent diameter and intertwine with each other. The results show that the nanofiber diameter distributions of both the PVA/HA and PCL/PEO layers are approximately normal. Meanwhile, the average fiber diameter of the PVA/HA layer is 0.42 ± 0.15 μm, whereas that of the PCL/PEO layer is 0.78 ± 0.23 μm. Comparatively, the fiber diameter of the PCL/PEO layer is larger.

**Figure 3:**
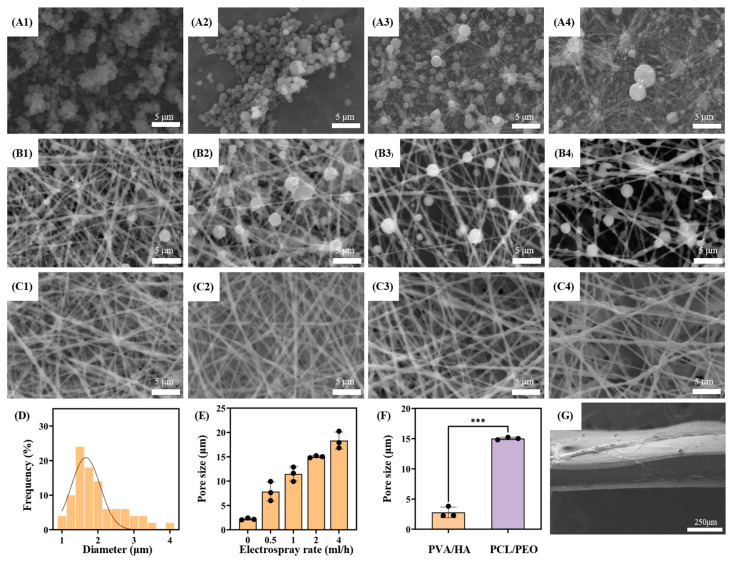
(**A**) SEM images of electrosprayed PVA (0588) microspheres at varying PVA solution concentrations: 3% *w*/*v* (**A1**), 6% *w*/*v* (**A2**), 9% *w*/*v* (**A3**), and 12% *w*/*v* (**A4**). (**B**) SEM micrographs of composite patches with varying electrospraying feed flow rates: 0.5 mL/h (**B1**), 1 mL/h (**B2**), 2 mL/h (**B3**), and 4 mL/h (**B4**). (**C**) SEM micrographs of composite patches whose microspheres are washed out with varying electrospraying feed flow rates: 0.5 mL/h (**C1**), 1 mL/h (**C2**), 2 mL/h (**C3**), and 4 mL/h (**C4**). (**D**) Diameter distribution of PVA (0588) microspheres with 6% *w*/*v* concentration. (**E**) Pore size of composite patches whose microspheres are washed out with varying electrospraying flow rates from 0.5, 1, 2, and 4 mL/h. (**F**) Average pore size of the PVA/HA and PCL/PEO layers. (**G**) The macrograph of cross-section of the composite patches. (E: n = 3, F: n = 3), with statistical significance indicated by *** *p* < 0.001.

The microscopic morphology of the PVA (0588) microspheres at various concentrations is displayed in Figure 3A. Entanglement between the individual chains does not exist at lower polymer concentrations. However, the polymer chains start to entwine and form thread-like fibers when the concentration reaches a critical overlap threshold [23]. Specifically, at the same electrospray feed rate, PVA (0588) forms spherical microspheres at a concentration of 6% *w*/*v*, while at 9% *w*/*v*, some microspheres transition into thread-like fibers. As shown in Figure 3A, the size of the PVA (0588) microspheres increases with concentrations up to 9% *w*/*v*. At this concentration, some fibers begin to form, while at 12% *w*/*v*, only a few microspheres are observed. To maximize the average pore size of the nanofiber scaffolds, a PVA (0588) concentration of 6% *w*/*v* was selected for this study.

As shown in Figure 3B,C, PCL/PEO fibers and PVA (0588) microspheres are produced simultaneously to form a composite membrane, with the PCL/PEO fiber to PVA (0588) microsphere ratio being controlled by adjusting the electrospray flow rate. The SEM images display that although the proportion of PVA (0588) microspheres varies, the diameter of the PCL/PEO fibers remains consistent. A random deposition pattern of the PVA (0588) microspheres and PCL/PEO nanofibers on the collector is observed, as illustrated in Figure 3B, where the fibers are interspersed with the embedded PVA (0588) microspheres. Furthermore, the SEM micrographs reveal the varying mixing ratios of the PCL/PEO fibers and PVA (0588) microspheres, and more aggregated PVA (0588) microspheres can be produced by increasing the electrospray flow rate. Thus, as shown in Figure 3C, the pore size within the nanofiber membrane increases with the addition of more embedded PVA (0588) microspheres.

As shown in Figure 3D, when the concentration of PVA (0588) is 6%, the electrosprayed microspheres exhibit an average diameter of 1.956 ± 0.681 μm, following a normal distribution. Additionally, Figure 3E illustrates that the purely electrospun nanofiber scaffold has an average pore size of 2.29 ± 0.21 μm. At an electrospray feed rate of 0.5 mL/h, the average pore size reaches 7.85 ± 1.98 μm, further increasing to 11.478 ± 1.476 μm at 1 mL/h, 15.02 ± 0.30 μm at 2 mL/h, and 18.33 ± 1.75 μm at 4 mL/h. The results indicate that the porosity and average pore size of the PCL/PEO nanofiber scaffolds increases with the electrospray feed rate. However, at 4 mL/h, a stable Taylor cone cannot consistently form, which may lead to the generation of large droplets that fail to produce microspheres due to the unstable evaporation times. Therefore, a feed rate of 2 mL/h was selected for the electrospraying.

In addition, as shown in Figure 3G, the SEM images of the composite patch cross-section reveal a well-bonded three-layer structure, with intertwined fibers and scaffolds and no significant delamination. Hence, the results indicate that the average pore size of the anti-adhesion layer is 2.79 ± 0.89 μm, while the growth-promoting layer exhibits a pore size of 15.02 ± 0.30 μm. Comparatively, the growth-promoting layer can provide a microenvironment that is more conducive to cell growth and migration. Furthermore, the composite patch demonstrates effective bonding between the layers, ensuring that they do not easily separate after implantation.

### 3.2. FTIR Analysis

**Figure 4:**
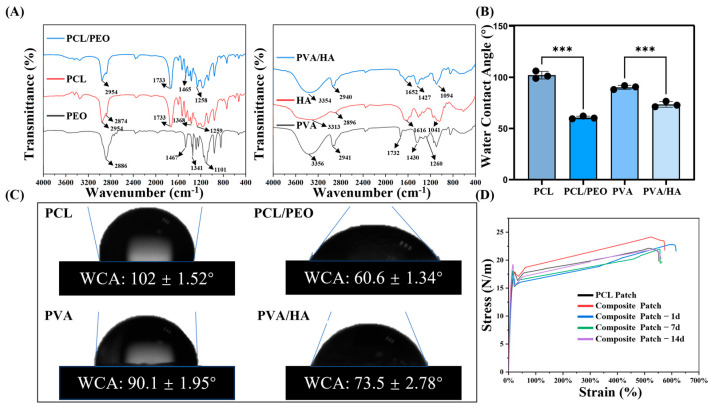
(**A**) The FTIR spectra of PCL, PEO, PCL/PEO, PVA, HA, and PVA/HA. (**B**,**C**) The WCA of PCL, PCL/PEO, PVA, and PVA/HA. (**D**) The tensile stress–strain curves of the PCL patch, composite patch and composite patch in a simulated in vivo environment for 1 day, 7 days and 14 days under the fixed tensile condition. (B: n = 3; D: n = 3), with statistical significance indicated by *** *p* < 0.001.

The FTIR spectra of the PCL, PEO, and PCL/PEO samples are shown in Figure 4A. As illustrated in the first figure in Figure 4A, the characteristic peaks of PCL include CH_2_ stretching at 2954 cm^−1^, C=O carbonyl stretching at 2874 cm^−1^, C–O and C–C stretching at 1733 cm^−1^ within the crystalline phase, and symmetric C–O–C stretching at 1259 cm^−1^. For PEO, characteristic peaks are also present, including C–H stretching at 2886 cm^−1^, a C–H bending of the alkane group at 1467 cm^−1^, and O–H bending at 1341 cm^−1^. In the PEO/PCL mixture, the C–H bending alkane group of PEO appears at 1465 cm^−1^, while PCL contributes CH_2_ stretching, C–C stretching, and symmetric C–O–C stretching at 2954 cm^−1^, 1733 cm^−1^, and 1258 cm^−1^, respectively [39,40].

The FTIR spectra of the PVA, HA, and the PVA/HA mixture are presented in the second figure in Figure 4A. In the PVA spectrum, O–H stretching, C–H stretching, and C–H bending are detected at 3356 cm^−1^, 2941 cm^−1^, and 1430 cm^−1^, respectively. Furthermore, the weak bands at 1732 cm^−1^ and 1260 cm^−1^ are attributed to C=O stretching and methyl(-COO-CH_3_) stretching vibrations originating from the partially hydrolyzed vinyl acetate groups of PVA. The absorption bands of HA appear at 3313 cm^−1^ due to NH and OH stretching, at 2896 cm^−1^ for C–H stretching, at 1616 cm^−1^ for C=O vibrations in amide II, and at 1041 cm^−1^ for C–O stretching. For PVA/HA, both the PVA absorption bands and the HA amide group’s characteristic peaks are visible [41].

In summary, the PCL/PEO and PVA/HA blends display all the functional groups of their respective components, with no new functional groups detected, suggesting that the interaction between these mixtures is purely physical, with no evidence of chemical reactions.

### 3.3. Wettability Analysis

Hydrophilicity affects the biocompatibility of biomaterials, the hemocompatibility, and the cell attachment [42]. Increased hydrophilicity positively influences the biocompatibility of the biomaterials, thereby enhancing the cell attachment, proliferation, and differentiation on the material surface [43]. The WCAs of the PVA/HA and PCL/PEO materials are shown in Figure 4B,C. The WCA of PCL is 102.0 ± 1.52°, which changes to 60.6 ± 1.34° with the addition of PEO, and the WCA of PVA is 90.1 ± 1.95°, which becomes 73.5 ± 2.78° after the addition of HA. The results suggest that the addition of hydrophilic materials PEO and HA could significantly improve the hydrophilicity of the raw materials PCL and PVA, respectively [44], which is beneficial for enhancing the biocompatibility of the materials, promoting the cell adhesion and proliferation of cells on the patches.

### 3.4. Mechanical Property Evaluation

The main role of abdominal wall defect repair patches is to resist the internal pressure in the human body and to support the new tissues, so the mechanical properties are one of the critical parameters for evaluating abdominal wall repair patches [45]. Studies have shown that the ideal tensile strength of abdominal wall repair patches should be at least 16 N/cm [46]. In order to investigate the changes in the mechanical properties of the Janus composite patches in a simulated in vivo environment, in addition to setting up a PCL group and a composite patch group, this study also set up an experimental group for the mechanical properties of the composite patches that were placed in a simulated in vivo environment for 1 day, 7 days and 14 days. This was achieved by placing the composite patches in PBS solution at 37 °C to simulate the in vivo environment and assessing the changes in their mechanical properties over time. The stress–strain curves from the fixed tensile tests are illustrated in Figure 4D. The tensile strength of the PCL patch was measured at 21.10 ± 2.51 N/cm, which met the mechanical standard of 16 N/cm. The composite patch exhibited a slightly higher tensile strength of 23.58 ± 1.00 N/cm, also meeting the requirements for abdominal wall repair patches. In addition, the mechanical strengths of the Janus composite patches were 22.41 ± 1.37 N/cm, 21.48 ± 0.34 N/cm, and 21.49 ± 0.55 N/cm, respectively, after being placed in a simulated in vivo environment (37 °C PBS solution). The results showed that the patches showed a slight decrease in strength on the first day in the wet environment, after which the mechanical properties stabilized. Only minor changes occurred over 14 days, and the strength at all the time points remained higher than the minimum standard required for abdominal wall repair. These results suggest that the composite manufacturing process maintains the mechanical properties of PCL patches while providing additional reinforcement.

### 3.5. In Vitro Cell Experiments

#### 3.5.1. Adhesion Analysis

Three groups of experimental subjects were established for the cellular studies: PVA/HA patches, PCL/PEO patches, and PCL patches (control group).

**Figure 5:**
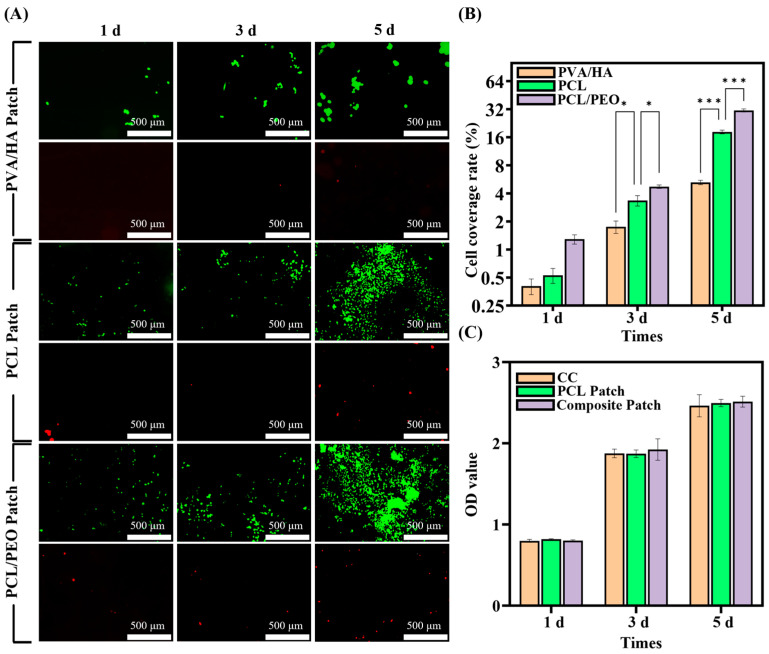
(**A**) Images of live/dead cells in the PVA/HA patch group, PCL patch group, and PCL/PEO patch group (live cells: green color, dead cells: red color). (**B**) The coverage rate of fluorescent cells on the surface of the patches was calculated after 1, 3, and 5 days of culture. (**C**) CCK-8 assays results after 1, 3, and 5 days of culture on HUVECs. (B: n = 3; C: n = 6), with statistical significance indicated by * *p* < 0.05, *** *p* < 0.001.

HUVECs grown on the three scaffolds are shown in the live/dead fluorescence pictures in Figure 5A at three different times. Over time, the number of living cells rose in all the groups while the number of dead cells stayed relatively low. Additionally, the fluorescent cell coverage was assessed, revealing that on the first day, the cell coverage rates for PVA/HA, PCL, and PCL/PEO were 0.40 ± 0.08%, 0.53 ± 0.10%, and 1.29 ± 0.15%, respectively. While there were slight differences between the groups, these were not obviously significant. However, the cell coverage of the patches appeared significant differences on the third and fifth days, with the PCL patches exhibiting coverage of 3.35± 0.43% and 18.23 ± 0.83%, respectively, while the PVA/HA patches showed coverage of 1.75 ± 0.27% and 5.24 ± 0.29%, respectively. These results suggest that the PVA/HA layer may not be conducive to cell proliferation and adhesion. In contrast, the cell coverage of the PCL/PEO patch was 4.72 ± 0.20% and 31.06 ± 1.19% on the third and fifth day, respectively, which shows that this layer favors cell proliferation and adhesion. The experiments show that the growth-promoting layer PCL/PEO in the composite patches had excellent biocompatibility, which was beneficial for tissue repair. On the contrary, the anti-adhesion layer PVA/HA had an inhibitory effect on cell adhesion and proliferation, and it could play a role in preventing adhesion.

#### 3.5.2. Cell Proliferation Assay

Cell proliferation is a crucial physiological function that underpins cell growth, reproduction, and heredity. In this study, the CCK-8 assay was used to detect the effect of the patch materials on cell proliferation. Figure 5C indicates the CCK-8 optical density (OD) values of the blank control (CC), PCL patch, and Janus three-layer composite patch at 1, 3, and 5 days. The data show that there were no appreciable variations in the OD values across the three groups. On the third and fifth days, however, the composite patch group showed somewhat higher OD values, which may indicate an impact on cell growth. While HA has been shown to promote cell proliferation, its impact may be limited due to its low concentration. Additionally, the experimental findings verified that all the materials tested were non-toxic.

#### 3.5.3. Cell Morphology Assay

**Figure 6:**
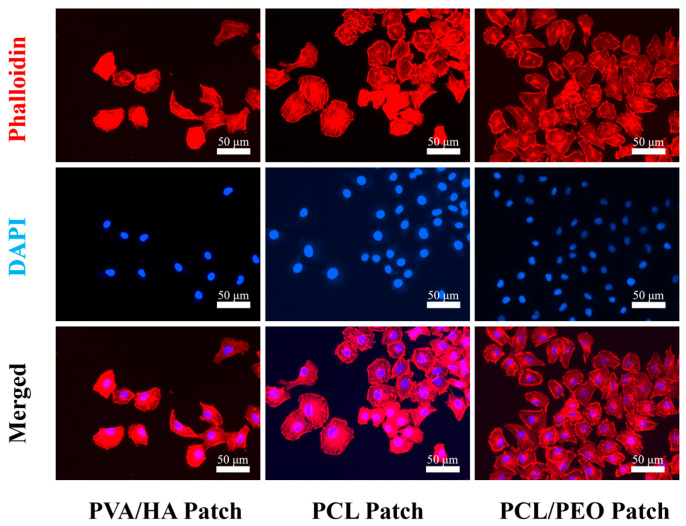
Images of the nucleus (blue) and cytoskeleton (red) of HUVECs grown on the PVA/HA patch, PCL patch and PCL/PEO patch for three days were captured using fluorescence.

The cytoskeletal staining experiments further investigated the morphology of the HUVECs on the different layers of the composite patch: PCL/PEO, PCL, and PVA/HA (Figure 6). The cells proliferated and aggregated on all the layers, forming tight intercellular connections, with clear visibility of the actin filaments and nuclei. These observations suggest that the HUVECs had normal adhesion, extension, and proliferation ability on all the layers of the composite patch, demonstrating that the composite patch was non-toxic to the cells and had good biocompatibility.

### 3.6. In Vivo Animal Experiment

**Figure 7:**
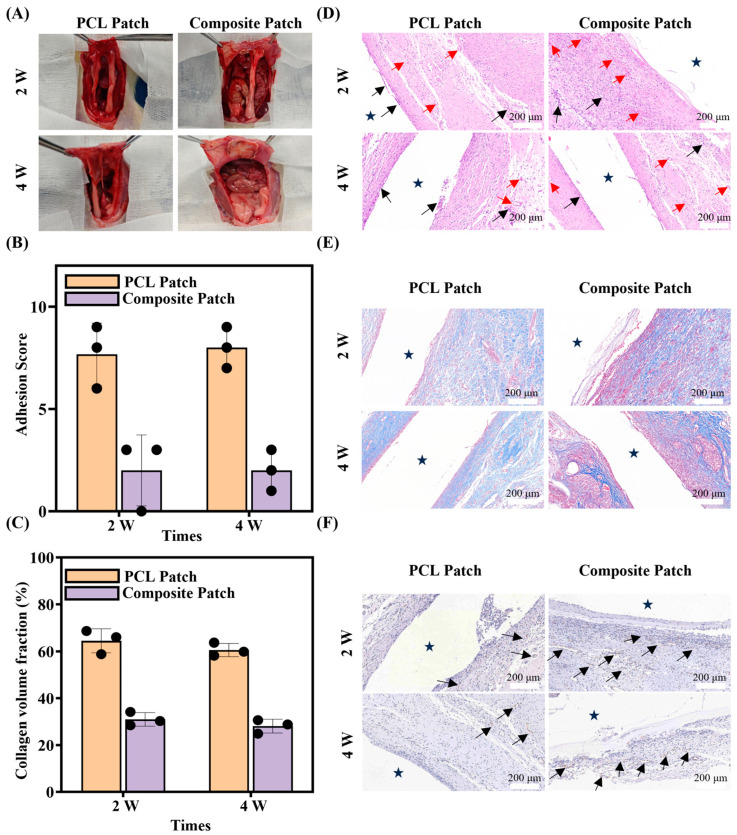
(**A**) Images of the wound status in the PCL patch group and composite patch group after 2 and 4 weeks. (**B**) Adhesion score 2 and 4 weeks after implantation (n = 3). (**C**) Collagen volume fraction (CVF) used to analyze the fibroplasia of the regenerative tissue in the PCL patch group and composite patch group quantitatively (n = 3). H&E (**D**), Masson (**E**), and CD31 (**F**) staining microscopic sections of the PCL patch group and composite patch group after 2 and 4 weeks (black arrow: inflammatory cells, red arrow: neovascularization).

The PCL patch (control group) and composite patches were used in the in vivo experiments. During the experiment, no rats died in either group. The repair outcomes for the PCL and composite patches after 2 and 4 weeks of implantation are shown in Figure 7A. By the second week, notable adhesion was observed in the PCL group, where the patch was covered by intestines and other internal organs. In contrast, the composite patch demonstrated significantly reduced adhesion. By the fourth week, adhesion with the composite patch had further diminished, likely as a result of the partial degradation of the PVA/HA layer, which created separation between the organs and the patch. In addition, no significant damage or degradation was found in the two patch groups, which proves that the patch can provide continuous and stable mechanical support in vivo. The quantitative scoring of the adhesion in each group is shown in Figure 7B. The PCL patch had an adhesion score of 7.7 at 2 weeks and 8 at 4 weeks, whereas the composite patch maintained a low adhesion score of 2 at both time points. The experimental results suggest that the PVA/HA layer in the composite patches had excellent anti-adhesion properties and could act as an effective physical barrier to create favorable conditions for defect repair.

In this study, H&E staining, Masson staining, and immunofluorescence staining were used to assess the wound healing of the two groups of patches at two and four weeks post-implantation, where the patch locations have been marked (shown by the star pattern). Extensive neovascularization around the PCL/PEO layer of the composite patch (shown by red arrows) was observed in the H&E staining results, as presented in Figure 7D, whereas severe inflammation was detected around the PCL patch at both the second and fourth weeks (shown by black arrows). This result indicates that the composite patch had a superior repair effect compared to the PCL patch.

The Masson staining was further analyzed for the tissue composition at the wound healing site, with the results presented in Figure 7E. In the Masson staining images, collagen fibers appear in blue, while myofibers are shown in red. The composite patch group exhibited greater myofibrillar tissue formation with relatively less collagen deposition at the defect site. In contrast, the PCL patch group showed increased collagen fiber formation with comparatively lower muscle fiber generation. As shown in Figure 7C, the CVF analysis results of both patch groups at two and four weeks indicate that at the second week, the CVF value of the PCL patch group was 64.47 ± 5.08%, while that of the composite patch group was 30.95 ± 2.91%. In the fourth week, the CVF value in the PCL patch group was 60.58 ± 2.84%, compared to 28.10% ± 2.90% in the composite patch group. The composite patch consistently showed significantly lower CVF values than the PCL patch at both the second and fourth weeks, and there was little difference observed within each group over time.

The immunofluorescence staining results for CD31 are shown in Figure 7F, where neovessels appear as elongated brown oval bar structures (indicated by black arrows). Neovascularization was observed in both patch groups, with a higher degree observed around the composite patch. This suggests that the composite patches were favorable for wound healing compared with the PCL patches. In conclusion, the composite patches exhibited effective anti-adhesion properties and promoted tissue defect repair, demonstrating favorable outcomes for abdominal wall defect repair.

## 4. Discussion

Abdominal wall defects are a common surgical condition, and tension-free hernia repair using polymer abdominal wall patches is currently the gold standard for the treatment of abdominal wall defects. However, due to the non-degradable and limited biocompatibility of polymer abdominal wall patches, implantation may trigger foreign body reaction, abdominal adhesions, and poor wound healing, so researchers are still exploring more ideal patches for abdominal wall repair [3,4,5]. In this study, a Janus three-layer composite patch for abdominal wall defect repair was developed using 3D printing, electrostatic jetting, and electrospinning techniques, and its physicochemical properties, biocompatibility, and tissue repair ability were evaluated. The results showed that the Janus three-layer composite patch prepared in this study exhibited excellent results in terms of both the mechanical and biological properties.

The pore size of nanofibrous scaffolds directly affects cell behavior. The small pore size of the scaffolds obtained by conventional electrospinning technology can effectively prevent cell adhesion and reduce tissue adhesion, but the too small pore size limits tissue integration [23]. To address this problem, this study combined the electrostatic spraying and electrospinning processes to form a uniform microporous structure inside the fibrous scaffolds, thus improving their pore properties. The experimental results showed that this method can effectively increase the average pore size of the scaffolds and improve the cell adhesion, proliferation and migration.

In terms of the mechanical properties, mechanical tests showed that the tensile strength of the patch (23.58 N/cm) was significantly higher than the minimum strength required for abdominal wall patches (16 N/cm) reported in the literature [45]. Meanwhile, the patch maintained good mechanical stability in the simulated in vivo environment. This is mainly attributed to the fact that the PCL consists of hydrophobic aliphatic hexane units that are linked to ester groups, thus limiting water absorption [47]. This property was also verified in vivo experiments, where the Janus patch showed good stability without rupture after four weeks of implantation, further demonstrating its mechanical reliability in physiological environments.

In terms of promoting tissue repair, PCL/PEO layers exhibit excellent biocompatibility. Due to its excellent rheological and viscoelastic properties, PCL is widely used as a biomedical material [24,25]. However, its high hydrophobicity makes it difficult for cells to adhere, which in turn affects tissue integration [26]. In this study, we improved the hydrophilicity of the PCL layer by introducing PEO and modulated its degradation rate to optimize the biocompatibility of the patch. The experimental results showed that the introduction of PEO significantly improved the hydrophilicity of PCL and enhanced the cell adhesion on its surface. In in vitro experiments, the PCL/PEO layer of the Janus patch was able to effectively promote the adhesion and proliferation of endothelial cells; histological analysis further confirmed that in the in vivo experiments, the Janus patch group showed more neovascularization and myofibrillar tissues as compared to the control group, suggesting that the layer could effectively promote neovascularization and improve the tissue integration ability. In addition, the Janus patch induced a significantly lower inflammatory response during tissue repair. Compared to conventional PP patches that exhibit a wide range of inflammatory responses, the Janus patches exhibited a milder host immune response [48,49]. This result is consistent with the findings that nanofiber scaffolds can reduce foreign body reaction and promote tissue repair, as reported in existing studies [18,50]. The in vitro experiments further verified that the large pore size patch facilitated cell growth and infiltration, which is consistent with the findings of Liang et al. and supports the advantages of a PCL/PEO layer in tissue repair [11].

In terms of the anti-adhesion function, the Janus patch is composed of a dense PVA/HA electrospun membrane on the visceral side, which forms a physical barrier to prevent direct contact between the abdominal organs and the patch surface, and HA, due to its excellent lubricating properties, can inhibit the adhesion of proteins and cells on the surface and reduce the occurrence of postoperative adhesions [28]. Sodium hyaluronate and its derivatives have been shown to be effective in reducing the incidence of abdominal adhesions as an anti-adhesion material [51,52]. In this study, we improved the spinnability of HA by co-electrospinning HA with PVA to obtain uniform and dense nanofibrous membranes, which further enhanced the structural integrity and anti-adhesion properties of the anti-adhesion layer. The Janus patch significantly reduced the endothelial cell adhesion in in vitro experiments, and the in vivo experimental results showed that it did not trigger significant visceral tissue adhesion after implantation.

Overall, a Janus three-layer composite patch with a combination of anti-adhesion, pro-healing, and long-term stability was prepared in this study, and its excellent biocompatibility and tissue integration ability were systematically verified. Future studies will further optimize the pore structure of the patch to promote the clinical translation of this technology and provide new solutions for abdominal wall defect repair.

## 5. Conclusions

In this study, a Janus three-layer composite patch was prepared based on 3D printing, electrostatic spinning and electrospray technology, whose electrostatic spinning scaffolds near the abdominal muscle side could promote tissue growth, and the electrostatic spinning scaffolds near the visceral side could effectively prevent cell adhesion. Physicochemical tests showed that the patch had good mechanical properties (23.58 N/cm) and hydrophilicity (73.5° for the anti-adhesion layer and 60.6° for the growth-promoting layer), while the anti-adhesion layer had a dense structure (average pore size of 2.79 μm) and the growth-promoting layer had a loose and porous structure (average pore size of 15.022 μm). In vitro cellular experiments suggested that the anti-adhesion layer of the patch could reduce cell migration, and the growth-promoting layer could effectively promote cell proliferation, while the patch had good cytocompatibility. In vivo animal experiments showed that the composite patch could reduce tissue adhesion and promote tissue repair. In conclusion, this patch has great potential for the future repair of abdominal wall defects.

## Data Availability

The data that support the findings of this study are available from the corresponding author (Haiguang Zhang) upon reasonable request.

## Abbreviations

The following abbreviations are used in this manuscript:

PP	polypropylene
PET	poly(ethylene terephthalate)
PTFE	polytetrafluoroethylene
SIS	small intestinal submucosa
PVA	polyvinyl alcohol)
ECM	extracellular matrix
PLGA	poly(lactic-co-glycolic acid)
PCL	polycaprolactone
PEO	Poly(ethylene oxide)
HA	sodium hyaluronate
SP	soy peptide
DCM	dichloromethane
DMF	N,N-dimethylformamide
AA	acetic acid
SEM	scanning electron microscope
WCA	water contact angle
HUVECs	human umbilical vein endothelial cells
ANOVA	one-way analysis of variance
OD	optical density
